# Inuit interpreters engaged in end-of-life care in Nunavik, Northern Quebec

**DOI:** 10.1080/22423982.2017.1291868

**Published:** 2017-03-07

**Authors:** Shawn Renee Hordyk, Mary Ellen Macdonald, Paul Brassard

**Affiliations:** ^a^ Department of Psychoéducation, Université de Montréal, Montreal, Quebec, Canada​​​​; ^b^ Oral Health and Society Research Unit, Faculty of Dentistry, McGill University, Montreal, Quebec, Canada​​​​; ^c^ Department of Medicine and Epidemiology, Biostatistics and Occupational Health, McGill University, Montreal, Canada; ^d^ Centre for Clinical Epidemiology, Jewish General Hospital, Montreal, Canada

**Keywords:** Interpretation, Inuit, Nunavik, Moral distress, End of life care

## Abstract

**Background:** Inuit interpreters are key players in end-of-life (EOL) care for Nunavik patients and families. This emotionally intensive work requires expertise in French, English and Inuit dialects to negotiate linguistic and cultural challenges. Cultural differences among medical institutions and Inuit communities can lead to value conflicts and moral dilemmas as interpreters navigate how best to transmit messages of care at EOL.

**Objectives:** Our goal was to understand the experience of Inuit interpreters in the context of EOL care in Nunavik in order to identify training needs.

**Design:** In the context of a larger ethnographic project on EOL care in Nunavik, we met with 24 current and former interpreters from local health centres and Montreal tertiary care contexts. Data included informal and formal interviews focusing on linguistic resources, experiences concerning EOL care, and suggestions for the development of interpretation training.

**Results:** Inuit working as interpreters in Nunavik are hired to provide multiple services of which interpretation plays only a part. Many have no formal training and have few resources (e.g. visual aids, dictionaries) to draw upon during medical consultations. Given the small size of communities, many interpreters personally know their clients and often feel overwhelmed by moral dilemmas when translating EOL information for patients and families. The concept of moral distress is a helpful lens to make sense of their experience, including personal and professional repercussions.

**Conclusions:** Inuit interpreters in Nunavik are working with little training yet in context with multiple linguistic and cultural challenges. Linguistic and cultural resources and focused training on moral dilemmas unique to circumpolar contexts could contribute to improved work conditions and ultimately to patient care.​​​​

## Introduction

Health care often occurs in multicultural contexts in which health care providers and their patients are approaching illness with differing cultural lenses and illness ideologies.[1] Interpreters are key players in bridging these differences. Interpreters are essential to health literacy of patients as they ensure that patients receive basic health information through which they are enabled to make appropriate health decisions.[2,3] Interpreters’ success in performing their tasks rests on institutional commitments to address both linguistic communication and cultural approaches in patient care.[4] The provision of interpreter training ensures that communication errors are decreased, that health services are more accessible for patients and families, and that patient and family understanding of diagnosis, prognosis and symptom-management are greater; doing so also ensures health providers are more satisfied with their work.[5,6]

## Review of the literature

The success of an interpreter-mediated medical encounter depends on the communicative skills and experiences of the interpreter and the relationships among those present in the encounter.[1] Hsieh [1] has argued that interpreter training must go beyond traditional values related to accuracy, faithfulness and neutrality; training must also understand the unique demands faced by interpreters who arrive to the encounter with divergent competencies and pre-existing relationships to patients. Further, within the encounter are health care providers whose capacities to engage in interpreter-mediated, intercultural dialogue may greatly vary. Unfortunately, “interpreters” are often not trained adequately to integrate these dynamics. In health contexts, family members, friends, and others providing informal interpretation services are known as “ad hoc” interpreters.[7] Though ad hoc interpreters may be allies in care due to their relational proximity to the patient,[5] they may not always be prepared for the level of interpretive responsibility required in health care.[7–9] Indeed, ad hoc arrangement may be at times “ethically indefensible,” as errors in interpretation may seriously impact patient health outcomes.[10]

In the context of end of life (EOL) care, trust built through communication between the patient and the treating physician is essential [11] and is thus a key goal in the interpreter-mediated medical encounter. Linguistic and cultural barriers, however, can serve to “engender frustration and reciprocal mistrust” (p. 4)[12]​​​​ between patient and physician. Misunderstandings in communication affect patient access to care, quality of care, patient capacity to provide informed consent, patient understanding of treatment directives or preventative measures, and patient adherence to treatment.[10,12,13] For patients diagnosed with a life threatening illness, patient-centred communication is therefore of “paramount importance”.[14] For interpreters engaged in EOL discussions, however, these conversations are less satisfying and more stressful than those concerning other health issues due to their significant cultural implications and intense emotional content.[15,16] Furthermore, interpreters often lack training in how to engage in EOL care dialogue, such as how to deliver bad news, to discuss code status and to notify families of the death of a loved one.[16]

### Interpreter context: Nunavik, Quebec

Quebec’s Charter of the French language stipulates that all residents must have access to health services in French.[17] In addition, it states that Inuit must be given opportunity to preserve and develop their own language and culture.[18] Neither the Charter of French Language nor Quebec’s Act Respecting Health Services and Social Services [19] addresses the question of developing adequate interpretation services for health care providers working with Inuit patients and families. Notably, a 2001 Health Canada report [18] concluded that there was a lack of consensus on the interpreter role in health services, and an absence of interpreter perspectives concerning the complexities of their roles. Little is known about the emotional impact of work on interpreters and the challenges they face in negotiating institutional cultures and ethnic groups.[18]

Unlike the large hospital settings where some interpreter experiences have been explored, interpreters working in Nunavik, Northern Quebec, often belong to, and work in, remote, isolated communities. Nunavik is divided into 14 communities ranging from approximately 250 to 1250 persons.[20] Each community has an outpatient health centre staffed by a minimum of two nurses. Larger communities may have onsite physicians as well as a larger cadre of nursing staff, including a home care nurse when needed. Two communities have inpatient hospitals providing limited care. A large majority of physicians and nurses working in Nunavik speak French as their primary language, the official language of the province of Quebec. In contrast, 95% of Nunavik’s residents speak Inuktitut as their mother tongue, with 20% communicating only in this language.[20] Three quarters of the region’s Inuit have a working knowledge of English with only a quarter (23%) with varying capacities to communicate in French.[20]

While examining the larger context of EOL care needs across the remote communities in Nunavik,[21] data from Inuit community members and health care providers revealed the critical role of interpreters in negotiating EOL care. Further, it became clear that the challenges faced by interpreters were significant. We believe grappling with these issues is essential for the development of EOL services in the region. In the literature, formal interpreters have been assigned a variety of titles including “translators”, “patient advocate”, and “cultural broker”.[22,23] Following the language of our participants, however, we have chosen to use the term “interpreter”. Interpreters working in Nunavik negotiate the language and cultural territories of patients and health care providers who speak French, English and/or Inuktitut. They are responsible for EOL conversations with family and community members, many with whom they interact on a personal level outside of the work environments.

## Methodology

Given the importance of understanding how sociocultural context affects care provision, for our larger study we used a focused ethnographic methodology.[24] Partners included the Nunavik Regional Health and Social Services Board, the two regional hospital centres, municipal leaders and school leaders. Data were collected between 2014 and 2015 during 14 weeks of field visits, ranging from one to six weeks, in the four Nunavik communities with the largest ageing populations. During these visits, researchers (two of the authors) conducted observation and informal and semi-structured interviews focusing on EOL care services and practices. We also conducted onsite interviews in the urban hospital centre in Montreal that receives Nunavik patients with complex palliative needs. In total, we spoke with 103 participants in the context of health centres, schools, municipal offices, family homes, churches, and community organisations; participants included nurses, physicians, social workers, interpreters and spiritual advisors. For this manuscript, we focus especially on the data addressing the interpreter role. Interviews and field notes were thematically coded and analysed using NVIVO software. Themes in the analysis concerning interpreters included dual roles, formal and informal tools and training, conflict and communication strategies, and contributions to care. These themes were drawn from interviews with interpreters, physicians, nurses and families. As per our ethnographic methodology, we contextualised the interview data with information from health reports, documentaries, news sources (e.g. Nunatsiaq online, a popular local source), books, art, museum exhibitions, and academic articles. The Institutional Review Board at McGill University approved our project.

## Findings

Overall, three major themes emerged related to interpreters: their diverse roles, their need for linguistic and cultural training and resources, and the ethical dilemmas they face concerning EOL care.

### The interpreter role

A 1960s documentary of a Nunavik family entitled “The Annanacks” [25] provides a portrait of the early interpreter in Nunavik. The interpreter was typically a male Inuit who had learned English by working alongside traders, missionaries, or whalers. He was a key player in economic transactions between the Inuktitut-speaking Inuit population and English-speaking government and industry representatives. When physicians began flying into settlements in the 1970s, it was often women living in communities who provided the majority of the health care interpretation. Their knowledge of English had largely come through residential school education and interactions with missionaries in the community, and their anatomical knowledge was largely from learned through hunting activities.

Since that time, it has become expected that Inuit health centre employees working in Nunavik with linguistic versatility fill multiple roles in the health care context. While interpretation services are part of these expectations, interpretation is always just one in a list of tasks an employee provides. For example, there are two local hospitals and one elderly care home​​​​ in Nunavik providing inpatient care. Those who provided interpretation services in these contexts are employed as “préposé en établissement nordique” – or “northern attendants”. These northern attendants speak English and/or French as well as Inuktitut. Their job description includes tasks such as cleaning, sterilising equipment, feeding and washing patients, as well as interpretation. Similarly, there are community health centres in each of the 14 communities, five offering additional home care services. Interpretive services are also provided in these settings, again by employees hired as northern attendants. Their work in these settings includes interpretation, as well as patient intake, measuring patient vital signs, sterilising equipment, cleaning, managing phones, setting appointments, organising files, and in some contexts accompanying home care nurses on their community rounds. Inuit employees also work in the large Montreal hospital centres where patients are sent. Again, they are employed in these settings as northern attendants. In these contexts they primarily provide interpretive services and patient accompaniment.

A management problem common across these diverse health contexts is low job retention and absenteeism of the northern attendants. When we asked for an explanation, we were given three primary reasons, two of which were specific to the task of interpretation and a third indirectly related: it is emotionally stressful to interpret difficult news and regulate conflict between patients, families and health care providers; there is little to no on-the-job linguistic training and supplementary interpreting resources; and the wages and benefits are not competitive compared to other local employment (e.g. with schools or the municipality). For example, northern attendants from Montreal hospitals told us that they had attained a high level of linguistic expertise through their years of experience, despite no on-the-job formal training. They were unhappy that they were not compensated for this experience, nor for the level of responsibility required in their interpretation tasks.

### The need for training and resources

Inuit interpreters described lack of linguistic training and resources as a major challenge in their work. While the anatomical knowledge they had attained through hunting, schooling and various media (e.g. television, radio and internet) had served them well, many described their own knowledge as inadequate. When medical terminology went beyond their knowledge, they turned to more experienced colleagues, as well as to dictionaries. Medical dictionaries are a limited resource, however, given that not all terms exist in Inuktitut; further, they often lack visual illustrations and thorough enough descriptions of body systems. In addition, not all interpreters had been trained to read in the two languages covered in the dictionary. Many interpreters described asking physicians and nurses to restate and clarify concepts; however, some were uncomfortable asking for this clarification, feeling it could be seen as unprofessional.

Responding to concerns of interpreters, the national Pauktuutit Inuit Women of Canada came together to publish an Inuit cancer glossary offered in five Inuktitut dialects.[26] In 2014, the Nunavut Culture and Heritage and Nunavut Department of Health [27] published a 400-page electronic anatomy glossary with visual illustrations of the body. While certainly useful, these resources were accessible to only some of the interpreters to whom we spoke. Further, interpreters told us that the most useful resources used visual illustrations and 3D models of the body that they could look at together with the patient and health care providers.

Interpreter training initiatives continue to be sparse and inconsistent for the northern attendants in Nunavik and in Montreal. As one interpreter stated, “We are a forgotten breed. We’re always needed but do not have the resources to do our job.” A few interpreters told us they had received some training in organisational skills (e.g. time-management, filing and computer skills) and, in recent years, some physicians had taken initiative to offer one- and two-day courses in anatomy and cultural communication to interpreters in Montreal. As one interpreter noted, “Compared to other professionals around them who are getting regular professional support, interpreters don’t receive regular training or have the structure to professionalise. No one has wanted to step forward to advocate for this.” Most interpreters with whom we spoke had received no training specific to the interpreter tasks of their job and they felt that this lack of training had a direct impact on patient trust. As observed by one experienced interpreter, “The new interpreters do not have enough training and are not wanted.” This lack was also openly noted by a new interpreter: “When I don’t understand the body parts, it’s kind of hard for me … the big words they have.”

Currently, there are no accredited post-secondary educational institutions in Nunavik, nor are there programmes in Montreal that provide interpreter certification or training. Several interpreters emphasised a need for additional linguistic training in anatomy and medical procedures inclusive of the various Inuit dialects. Further, they spoke of the need to adapt this training towards visual forms of communication and learning. They also spoke of the need for professional recognition of their services and wished for the opportunity to receive this recognition based on the training and experience they have had while working in the field.

### Moral dilemmas in EOL care conversations

In addition to wanting additional linguistic training and recognition generally, interpreters told us specifically that they needed training and support regarding the ethical decisions they encounter in dialogues between Inuit patients and southern-based health care providers about EOL care. Many described scenarios in which there were significant moral conflicts, especially when the values of the health care providers clashed with those of the communities, families and interpreters themselves. Interpreters described feeling caught between the expectations of the patient/family on one side and the health care providers/institution on the other. These encounters could produce moments during which no apparent “right” solution would emerge, leaving interpreters feeling isolated in the middle, an experience one described as “a very lonely experience.” Interpreters were left with deep emotional distress and without guidance in how to resolve such conflicts.

For example, interpreters are often expected by the health care team to transmit news of death and terminal diagnoses. Such tasks are traditionally assigned to community leaders and elders, known locally as *Tutsalukkaijiit*. These leaders and elders are recognised as having attained the life experience that equips them for the emotional weight of responding to family grief, and the wisdom to guide this process. Several interpreters described feeling overwhelmed when the health care providers assigned them such a task, feeling that they would be contravening local custom and social norms.

Similarly, when physicians were very direct in their prognosis concerning the length of time the patient had to live, interpreters felt conflicted about what to communicate to the patient: should they communicate this news directly to the patient or adapt the message to the patient’s familial and cultural context? Some interpreters, for example, believed an exact translation of the physician’s words was expected of them and thus provided this without exception. We noted, however, that such a direct approach appeared to be favoured by interpreters working in hospital centres and thus whose personal relationships with the patients were less intertwined then in smaller community contexts. In contrast, several described being guided by their own spiritual beliefs: they told patients and families that only God knew when someone would die and that humans could not predict this. One interpreter described how, when interpreting news to a patient who was also her friend, she was able to convey the gravity of the cancer diagnosis through gestures and vague statements without having to provide a literal translation. From the interpreter’s perspective, this had been well received by the patient. Another interpreter also described receiving positive feedback for her choice to be indirect:

A lot of people that I had interpreted for came back to thank me for not saying (they had cancer). They had a rough idea (of their prognosis), but because I kept reassuring them … “You can lengthen the time, you can still have a liveable functional life even though you are given that time. … Don’t have to be stuck in bed.” Giving them hope, it could lengthen …. Just giving them that hope. You just have to explain a little bit more. They really appreciate that.

Interpreters also described drawing on the experience of mentors, including more experienced interpreters and their own elder family members, in choosing how direct to be with patients about a terminal diagnosis. For example, a father had served as mentor for one interpreter, advising, “Never interpret word for word … Your fellow Inuk will be able to take it better.” She took his advice and described how she felt she had averted potential conflict in doing so. She also described other family influences: “I always put myself in their (patient and family) place and treat them how I would want to be treated. It might not always be the perfect way, but giving them dignity and respect and being discreet and stuff like that, those are the tricks I learned from my grandmother and my dad and of course my mother.”

Several interpreters chose how direct to be about a terminal diagnosis by thinking about the potential consequences of a physician’s message on the patient and family and themselves. Interpreters who adapted this approach had usually worked in Nunavik communities in which their daily lives overlapped with that of the patients and their families. One interpreter described how she had learned this lesson early on in her career:

Doctors from South said “just tell them he’s not going to live for a long time because he has sickness” … When I tried interpreting for the sick one, “you might die soon,” some people start to argue … give me a shock … “Why did you tell him? Why did you tell him he’s dying soon? Why did you have to say that?”

This interpreter transformed her own cultural authority as Inuit into a framework for providing interpretation services. In her words, “Doctors sometimes really do what they know. But from my side, I say, ‘You don’t know how the Inuit are doing’… Doctor doesn’t really understand (the Inuit). Just do what they know, what they learned.” This recognition that doctors only did what they were taught indicated that she did not judge them, nor did she allow them to usurp her cultural expertise as Inuit.

Interpreters are required to sign agreements to maintain patient confidentiality. Doing so can mean compromising their habitual self-care practices of reaching out to colleagues, friends or family members for emotional support when they are adversely impacted by their job. An interpreter described a moment where she was expected to communicate a terminal diagnosis to a patient. She recalled, “We know people from here. I felt heartbroken. I almost cried.” Another described a similar circumstance, “I thought I was going to be ok, but when we entered the house I got very sensitive so I was having a hard time to translate.” Intense emotions inherent to conversations concerning EOL care took their toll on interpreters. They needed support from colleagues, family and friends; however, due to the proximity of community relationships, this was often difficult to navigate without compromising patient confidentiality. As one interpreter reflected on her early months of practice: “At first I was having a hard time not to spit out the confidential … and I explained to my colleague, ‘How am I not going to talk?’”

One final area of conflict for interpreters relates to feeling caught between frustration on the part of the medical staff and the part of the patients and families. As one junior interpreter in Nunavik described, “The patients always complain to us. And the doctors and nurses, they complain to us too. We are all in the middle.” Interpreters were, at times, uncertain as to when to translate these emotional reactions between patients and health care providers, recognising that these emotional reactions did not serve a medical purpose and could potentially compromise the patient–health care provider relationship. Interpreters could also become targets of these frustrations themselves. In one instance, a health care provider, upset with a patient who missed an appointment, accused the interpreter of forgetting to schedule the appointment. Such questioning of interpreters’ competency also emerged when patients did not comply with treatment directives. In other instances, patients perceived physicians’ questions as intrusive and repetitive and got upset with the interpreter for what they felt was a violation of privacy; they questioned whether the interpreters had understood the first time the question was answered.

### Impact on nurses and physicians working in the context of EOL care

Our findings also revealed that physicians and nurses relied heavily on interpreters for cultural interpretations, as many felt that their training in discussing EOL care options in a culturally appropriate manner was lacking. Furthermore, they often did not know patients and families personally and thus lacked the trust foundation essential to EOL care dialogues. They were also often unaware of how factors in the context of care,[21] including prior bereavement and healthcare experiences, might impact patient and family perception, though they could sense tensions in the EOL care discussion. This, combined with their own feelings of isolation and fatigue at times led to nurses and physicians inadvertently placing additional stress on interpreters as they at times relied on interpreters not only for linguistic translations but also for patient background information, for cultural guidance and for emotional support. In light of this, we asked physicians, nurses and interpreters to discuss the strategies that they found effective in communication. ​​​​Summarised in Table 1, these strategies rely on humanising the relationship with not only patients, but interpreters as well; while taking into account the context of continuous pressure put on by health care demands.

**Table 1.** Communication guidelines in EOL care.

Working with Inuit health interpreters at end of life: guidelines for nurses and physicians
Time, time, take time – establish relational foundation for care, ensure clear communication
Keep sentences short
Learn about moral dilemmas faced by interpreters
Recognise that not all medical terms and procedures are translatable in Inuktitut
Explain why questions are asked of patient and families, sometimes repeatedly – avoid intrusion
Use visual illustrations with the patient and with interpreter at the same time
Shift the power dynamic when possible – recognise patient, family and interpreter knowledge, efforts and expertise
Welcome patient request for interpreters who appear to understand – interpreters may be requested as cultural mediators and providers of emotional support
Model through example – mentor caregiving techniques for families
Invite family to participate in care in home and hospital care settings
Explain difficult news to the interpreter ahead of time as this allows time for clarification and allows time for interpreter to address own emotions before speaking with patient
Explain the rationale of potentially controversial or emotionally loaded messages to interpreter – this equips the interpreter to communicate the spirit of a message and to address patient’s reactions more clearly
When using a telephone in interpretation, do so progressively rather than just one long conversation
Familiarise with worldviews, spiritual beliefs of patient and family – i.e. let patients know that even doctors cannot know the time of death
Attune to non-verbal messages looking at the patient, not the interpreter when speaking
Respectfully invite input from interpreters concerning how to communicate difficult messages, community contextual factors, and cultural knowledge
Be clear, provide potential scenarios concerning the potential impact of extraordinary life-sustaining treatment
Let go and let it be, attuning to patients who do not want life-saving interventions
Recheck the comprehension of patient – clarify when misunderstanding is not necessarily a problem with interpretation
Learn some words in the patient’s Inuktitut dialect and use them
Attend funerals when appropriate, asking if this is perceived as a gesture of support or an intrusion
Guide families and patients in communicating frustration – recognise causes of stress, set limits, clarify communication expectations
Seek help to address the social suffering surrounding patient care
Educate communities about medical procedures, dying process through public health announcements – radio, Facebook, video, illustrated brochures, in Inuktitut
(See [28])

## Discussion

To perform their tasks adequately, interpreters described needing linguistic, cultural, and communicative competence. An important distinction in the literature is between competency in *langue* – attention to words, and *parole* – the gestures, delivery and awareness of the cultural, social and historical contexts that give words and phrases their meaning.[29] Attention to the social norms of communication as well as grammatical correctness ensures a clinical encounter is not “derailed”;[29] this fact is well recognised by the Inuit interpreters with whom we spoke who had incorporated cultural knowledge into their interpretive approach in EOL care dialogues. Attention to *parole* led to conflict however, as this could mean changing a physician’s message. Interpreters found themselves making moral decisions concerning how to translate terminal diagnoses, sometimes without having a clear sense as to how to best make these decisions. This process was further complicated when lack of resources resulted in linguistic confusion.

## Implications

### Linguistic training and visual resources

Interpreters seeking to transmit messages between health care providers and patients and families described visual resources as the most effective means to communicate. Visual resources in the form of printed text have long been used by health professionals,[30] including those in circumpolar communities.[26,27] While significant progress has been made in providing resources to Nunavik interpreters, interpreters need more, especially to meet the ever-growing list of medical conditions and terminology they encountered in their daily roles. Further, anatomical drawings and textual translations are of limited use in the immediacy of the interaction between doctor and patient. Emerging digital resources may hold promise. One example is the three-dimensional education platform, the Biodigital Human,[2] with vivid graphics that can be accessed by health care providers while meeting with interpreters and patients. This resource may be limited in Nunavik contexts, however, given that wireless communication remains unreliable.

Training for interpreters in Nunavik can be difficult due to the geographic isolation of communities. Interpreters told us that local training was essential, however. Several recommended that this include professional certification based on experience that had acquired in the field. This recommendation corresponds to the initiatives of other countries who have adapted a “pragmatic, needs-based, and socially focused” [31] approach to translation in which training, testing, experience and recommendations from health care professionals are part of the certification process and in which interpreters are recognised for their contributions to health care.[31]

### Training in circumpolar contexts – addressing ethical dilemmas

Our findings may also be relevant for health educators seeking to equip Inuit interpreters to address the moral conflicts unique to their position. Interpreters are negotiating – *as they interpret* – how to alter and add to translations so that spiritual beliefs and cultural norms can be incorporated.[5,32] Yet, they have no formal training or guidance for this expertise. Based on the consistency with which interpreters identified a need for such training, and on the low retention and high absenteeism rates of interpreters in communities, we posit that the lack of such training may be significantly contributing to burnout as the literature suggests is the case in the nursing profession.[33,34]

The Canadian National Standard Guide for Community Interpreting Services [35] has produced a code of ethics emphasising the following: accuracy and fidelity in interpretation; confidentiality; impartiality; and respect for persons. As our findings confirm, codes of ethics such as these have been found insufficient to address the dilemmas faced by interpreters in the field.[5,36] Interpreter training in ethics tailored to the challenges inherent to the profession is key to quality care,[22,37] a finding echoed by our participants. Our data suggest that exploring the strategies that Nunavik interpreters are implementing to guide their ethical processes may be one way in which to tailor training for interpreters working in circumpolar contexts.

The interpreters with whom we spoke drew on four components as they sought clarity regarding moral dilemmas: (1) *Personal values*, informed by spiritual beliefs, and family and community communication practices; an example is an interpreter who stated, “Doctors … from my side, I say: ‘You don’t know how the Inuit are doing’” and instead adapted her interpretation to the patients’ needs; (2) *Formal and informal codes of ethics*: some adhered to confidentially agreements (formal codes) despite the personal consequences, while others were guided by family and community teachings regarding dignity and respect (informal codes) which could also result in professional conflicts; (3) *Institutional versus cultural norms*: for example, providing verbatim translation versus using cultural and spiritual guidance to adapt the translation; and (4) *Mentors*: without institutional support, interpreters sought guidance from external sources, drawing on colleagues and family members to guide their practice.

Together, these components begin to shape a framework for understanding how interpreters navigate moral dilemmas. We offer this framework as a starting point for a conversation on the training and mentoring needs of circumpolar interpreters as they negotiate the complexity of EOL care in family, community and institutional contexts. Engaging with interpreters in the field will further clarify and refine this framework. For example, we have not had the opportunity to delve into how self-care practices and the strength of community ties may become a factor in care provision.

### Training for health care physicians and nurses

Systemic changes such as improved cultural training for physicians and nurses providing EOL care and developing bereavement services for families (including interpreters) and increased care-giver–patient ratios would help to alleviate the stress placed on the interpreter seeking to address the parole and langue needs of patients, families, nurses and physicians.[28] In addition to these systemic changes, our findings indicate that physicians, nurses, and interpreters working in in circumpolar EOL care contexts can benefit from cultural training when working with Indigenous populations [38] as well as training concerning communication with medical interpreters in EOL care settings.[39] The findings addressed in this article suggest that understanding the role of interpreters in or from Northern Inuit communities entails a deeper understanding of how dual roles, moral dilemmas and limits posed by the Inuktitut vocabulary impact the effectiveness of an interpretation.

### Further research directions

The Nunavik Health Board has taken findings from this study and is in the process of developing and implementing training for physicians, nurses and interpreters who are providing care to Nunavik patients and families. Future research may identify whether these educational efforts improve the level of retention of interpreters and the increase satisfaction in communication of all who are involved in EOL care provision. Furthermore, the Canadian government is in the process of developing strategies to valorise the language of its many Indigenous communities within institutional contexts. We suggest that future research will support these efforts by identifying challenges interpreters face in other Canadian Indigenous communities, the strategies adapted in the face of these, and the proposed sustainable solutions.

## Conclusion

Nunavik interpreters are key players in EOL care provision, entering their role with diverse levels of expertise. Currently, no formal and consistent training programmes equip them to perform their tasks at an optimal level. Training that provides adequate tools in linguistic translation and moral dilemmas unique to circumpolar contexts, and which results in more formal professional status with associated working conditions, should ultimately contribute to improved patient care.

## References

[CIT0001] Hsieh E. (2016). Bilingual Health Communication: working with interpreters in cross-cultural care.

[CIT0002] Butow P (2015). Communicating with culturally and linguistically diverse patients in cancer care. Communicating Qual Saf Health Care.

[CIT0003] Sørensen K, Van den Broucke S, Fullam J (2012). Health literacy and public health: A systematic review and integration of definitions and models. BMC Public Health.

[CIT0004] Andrulis DP, Brach C (2007). Integrating literacy, culture, and language to improve health care quality for diverse populations. Am J Health Behav.

[CIT0005] Kaufert JM, Putsch RW (1997). Communication through interpreters in healthcare: ethical dilemmas arising from differences in class, culture, language, and power. J Clin Ethics.

[CIT0006] Silva MD, Genoff M, Zaballa A (2016). Interpreting at the end of life: a systematic review of the impact of interpreters on the delivery of palliative care services to cancer patients with limited english proficiency. J Pain Symptom Manage.

[CIT0007] Flores G, Abreu M, Barone CP (2012). Errors of medical interpretation and their potential clinical consequences: a comparison of professional versus Ad Hoc versus no interpreters. Ann Emerg Med.

[CIT0008] Hunt LM, de Voogd KB (2007). Are good intentions good enough? informed consent without trained interpreters. J Gen Intern Med.

[CIT0009] Flores G, Laws MB, Mayo SJ (2003). Errors in medical interpretation and their potential clinical consequences in pediatric encounters. Pediatrics.

[CIT0010] Segalowitz N, Kehayia E (2011). Exploring the Determinants of Language Barriers in Health Care (LBHC): toward a research agenda for the language sciences. Can Mod Lang Rev.

[CIT0011] Heyland DK, Dodek P, Rocker G (2006). What matters most in end-of-life care: perceptions of seriously ill patients and their family members. Can Med Assoc J.

[CIT0012] Surbone A (2009). Cultural competence in oncology: where do we stand?. Ann Oncol.

[CIT0013] Jacobs E, Chen AH, Karliner LS (2006). The need for more research on language barriers in health care: a proposed research agenda. Milbank Q.

[CIT0014] Lubrano di Ciccone B, Brown RF, Gueguen JA (2009). Interviewing patients using interpreters in an oncology setting: initial evaluation of a communication skills module. Ann Oncol.

[CIT0015] Kaufert JM, Putsch RW, Lavallée M (1999). End-of-life decision making among Aboriginal Canadians: interpretation, mediation, and discord in the communication of “bad news”. J Palliat Care.

[CIT0016] Schenker Y, Fernandez A, Kerr K (2012). Interpretation for discussions about end-of-life issues: results from a national survey of health care interpreters. J Palliat Med.

[CIT0017] Québec G (2002). Office Québécois de la Langue Française.

[CIT0018] Bowen S (2001). Language barriers in access to health care.

[CIT0019] Québec Gd (2016). Quebec’s act respecting health services and social services.

[CIT0020] Statistics Canada (2014). National Health Survey, Aboriginal Population Profile, Nunavik, Inuit region, Quebec.

[CIT0021] Hordyk SR, Macdonald ME, Brassard P.

[CIT0022] Baker M, Maier C (2011). Ethics in interpreter & translator training. Interpreter Trans Trainer.

[CIT0023] Kaufert JM, Koolage WW (1984). Role conflict among ‘culture brokers’: the experience of native Canadian medical interpreters. Soc Sci Med.

[CIT0024] Knoblauch H Focused ethnography. http://nbn-resolving.de/urn:.

[CIT0025] Bonnière R (1964). The annanacks. National Film Board.

[CIT0026] Pauktuutit Inuit Women of Canada (2013). Kaggutiq: inuit cancer glossary.

[CIT0027] Nunavut Culture and Heritage and Nunavut Department of Health (2014). Human anatomy glossary.

[CIT0028] Hordyk S, Macdonald M, Brassard P (2016). End of life care inuit in Nunavik Quebec. Submitted to the Nunavik regional board of health and social services.

[CIT0029] Gregg J, Saha S (2007). Communicative competence: a framework for understanding language barriers in health care. J Gen Intern Med.

[CIT0030] Randolph W, Viswanath K (2004). Lessons learned from public health mass media campaigns: marketing health in a crowded media world*. Annu Rev Public Health.

[CIT0031] Hlavac J (2013). A cross-national overview of translator and interpreter certification procedures. Int J Transl Interpreting Res.

[CIT0032] O’Neil JD, Cultural T (1989). Political context of patient dissatisfaction in cross-cultural clinical encounters: a Canadian inuit study. Med Anthropol Q.

[CIT0033] Whitehead PB, Herbertson RK, Hamric AB (2014). Moral distress among healthcare professionals: report of an institution-wide survey. J Nurs Scholarship.

[CIT0034] Pendry PS (2007). Moral distress: recognizing it to retain nurses. Nurs Econ.

[CIT0035] Health Care Interpretation Network (2007). The Canadian national standard guide for community interpreting services.

[CIT0036] Dysart-Gale D (2005). Communication models, professionalization, and the work of medical interpreters. Health Commun.

[CIT0037] Ono N, Kiuchi T, Ishikawa H (2013). Development and pilot testing of a novel education method for training medical interpreters. Patient Educ Couns.

[CIT0038] Johnston G, Vukic A, Parker S (2012). Cultural understanding in the provision of supportive and palliative care: perspectives in relation to an indigenous population. BMJ Support Palliat Care.

[CIT0039] Norris WM, Wenrich MD, Nielsen EL (2005). Communication about end-of-life care between language-discordant patients and clinicians: insights from medical interpreters. J Palliat Med.

